# *De Novo* Transcriptome Assembly and Analyses of Gene Expression during Photomorphogenesis in Diploid Wheat *Triticum monococcum*

**DOI:** 10.1371/journal.pone.0096855

**Published:** 2014-05-12

**Authors:** Samuel E. Fox, Matthew Geniza, Mamatha Hanumappa, Sushma Naithani, Chris Sullivan, Justin Preece, Vijay K. Tiwari, Justin Elser, Jeffrey M. Leonard, Abigail Sage, Cathy Gresham, Arnaud Kerhornou, Dan Bolser, Fiona McCarthy, Paul Kersey, Gerard R. Lazo, Pankaj Jaiswal

**Affiliations:** 1 Department of Botany and Plant Pathology, Oregon State University, Corvallis, Oregon, United States of America; 2 Molecular and Cellular Biology Graduate Program, Oregon State University, Corvallis, Oregon, United States of America; 3 Center for Genome Research and Biocomputing, Oregon State University, Corvallis, Oregon, United States of America; 4 Department of Crop and Soil Science, Oregon State University, Corvallis, Oregon, United States of America; 5 Institute for Genomics, Biocomputing and Biotechnology, Mississippi State University, Mississippi State, Mississippi, United States of America; 6 European Bioinformatics Institute, Hinxton, Cambridge, United Kingdom; 7 School of Animal and Comparative Biomedical Sciences, University of Arizona, Tucson, Arizona, United States of America; 8 USDA-ARS, Western Regional Research Center, Albany, California, United States of America; University of Delhi South Campus, India

## Abstract

**Background:**

*Triticum monococcum* (2n) is a close ancestor of *T. urartu*, the A-genome progenitor of cultivated hexaploid wheat, and is therefore a useful model for the study of components regulating photomorphogenesis in diploid wheat. In order to develop genetic and genomic resources for such a study, we constructed genome-wide transcriptomes of two *Triticum monococcum* subspecies, the wild winter wheat *T. monococcum ssp. aegilopoides* (accession G3116) and the domesticated spring wheat *T. monococcum ssp. monococcum* (accession DV92) by generating *de novo* assemblies of RNA-Seq data derived from both etiolated and green seedlings.

**Principal Findings:**

The *de novo* transcriptome assemblies of DV92 and G3116 represent 120,911 and 117,969 transcripts, respectively. We successfully mapped ∼90% of these transcripts from each accession to barley and ∼95% of the transcripts to *T. urartu* genomes. However, only ∼77% transcripts mapped to the annotated barley genes and ∼85% transcripts mapped to the annotated *T. urartu* genes. Differential gene expression analyses revealed 22% more light up-regulated and 35% more light down-regulated transcripts in the G3116 transcriptome compared to DV92. The DV92 and G3116 mRNA sequence reads aligned against the reference barley genome led to the identification of ∼500,000 single nucleotide polymorphism (SNP) and ∼22,000 simple sequence repeat (SSR) sites.

**Conclusions:**

*De novo* transcriptome assemblies of two accessions of the diploid wheat *T. monococcum* provide new empirical transcriptome references for improving Triticeae genome annotations, and insights into transcriptional programming during photomorphogenesis. The SNP and SSR sites identified in our analysis provide additional resources for the development of molecular markers.

## Introduction

Einkorn wheat is one of three cereal crops domesticated prior to 7000 B.C. that contributed to the Neolithic Revolution [1]. Stands of wild einkorn, subspecies *Triticum monococcum* ssp. *aegilopoides*, are extensive in rocky areas of southeastern Turkey [1]. Domesticated einkorn, subspecies *T. monococcum* L. ssp. *monococcum* L. (2n = 14) originated in the Karacadağ mountains of Turkey [2] and was widely cultivated during the Neolithic period. Domesticated einkorn differs from the wild accessions in possessing plumper seeds and tough rachis phenotypes that prevent seed shattering, a domesticated trait selected for avoiding loss of yield [3].

*T. monococcum*, carrying the representative diploid wheat A genome (A^m^A^m^), is closely related to *T. urartu* (A^u^A^u^), the donor of the A genome of cultivated hexaploid (AABBDD) wheat (*T. aestivum*) [4]. The genome size of *T. monococcum* is about 5.6 Gb, which is 12 times the size of the rice genome and 40 times the genome of the model dicot plant *Arabidopsis thaliana* [5]. However, in comparison to the ∼17 Gb genome size of common hexaploid wheat, the diploid *T. monococcum* offers relative simplicity and has been used extensively as a model [6]. The many existing wild populations of *T. monococcum* growing in their natural habitat have suffered little selection pressure and thus offer opportunities to study its diversity [7]. They also serve as a reservoir of useful alleles and traits, such as salinity tolerance [8] and disease resistance [9], [10], and thus have been utilized for generating genetic maps to facilitate comparative mapping [11] and map-based cloning of genes [12], [13]. Combining the sequence and positional information of the genes based on recently published barley (*Hordeum vulgare*) [14], *T. urartu* [15] and *Aegilops tauschii* [16], [17] genomes with the genetic tools and transcriptome-based resources available for *T. monococcum* reported herein will allow progress in future genetic studies in wheat and other closely-related species.

Light regulates a wide range of plant processes including seed germination, organ, cell and organelle differentiation, flowering [18]–[21] and metabolism [22]. The germination of a seed in the dark follows skotomorphogenesis (the growth of an etiolated seedling). Upon exposure to light, seedlings go through photomorphogenesis (greening) that is marked by chlorophyll biosynthesis, differentiation of protoplastids into chloroplasts, the initiation of carbon assimilation, elongation and thickening of the hypocotyl, and the activation of the shoot apical meristem leading to the development of the first true leaves [23]–[25]. Although the transition from skotomorphogenic to photomorphogenic growth has been well-documented in *Arabidopsis* [24], [25], the complex gene networks at the genome level controlling this developmental transition in wheat are not well understood.

In order to investigate and identify the complex transcriptional network associated with seedling photomorphogenesis in Einkorn wheat, we conducted Illumina-based transcriptome analyses (RNA-Seq) of two *T. monococcum* subspecies: DV92, a spring Einkorn accession of the cultivated *T. monococcum* ssp. *monococcum* collected in Italy and G3116, a wild winter Einkorn, *T. monococcum* ssp. *aegilopoides*, collected in Lebanon [11]. Computational analysis of the transcriptome data provided functional annotations to the gene models and gene families. We also identified gene loci harboring SSR and SNP sites and predicted their consequences on transcript structure, coding features and expression.

## Results

### Sequencing and *de novo* assembly of transcriptomes

A total of twelve cDNA libraries were created, six from each of the DV92 and G3116 accessions. These libraries represent three replicates prepared from dark-grown seedlings sampled eight days (8DD) after germination, and three replicates prepared from seedlings grown in the dark for eight days and then exposed to continuous light for 48 hours, sampled eleven days after germination (48LL). The sequencing of cDNA libraries from the 8DD and 48LL samples on the Illumina HiSeq 2000 platform generated 39.56 Gbp of nucleotide sequence from DV92 and 37.65 Gbp from G3116. *De novo* assemblies were performed using Velvet and Oases [26], resulting in a total number of 120,911 transcripts for DV92 and 117,969 transcripts for G3116 (≥200 bp in length; Table 1). The assemblies of each accession were created in a two-step process: first, two separate assemblies were generated from optimized 31 and 35 K-mer lengths; second, transcript isoforms were clustered to obtain discrete assemblies for DV92 and G3116, representing the total number of unique transcripts after merging. The quality of transcriptome assemblies was assessed with various statistical metrics including the overall number (coverage), average length and diversity of transcripts (the estimated number of discrete loci assembled), and via comparison with published, annotated genomes. The average length for DV92-derived transcripts was 1,847 bp; the average length for G3116-derived transcripts was 1,783 bp (Table 1). The overall frequency distributions of transcript lengths are similar to other *de novo* plant transcriptome assemblies [27]–[29] and similar to the overall distribution of barley and *T. urartu* gene lengths (Figure S1).

**Table 1:** Transcriptome assembly statistics.

Transcriptome assemblies	Total number of reads	Number of Transcripts	Largest sequence (bp)	Average length (bp)	Median length (bp)
**DV92-31 k-mer**	435,806,374	87,972	21,251	1633	1393
**DV92-35 k-mer**	435,806,374	82,185	13,427	1699	1460
**DV92 Merged**		**120,911**	**21,331**	**1847**	**1600**
**G3116-31 k-mer**	366,215,814	84,491	21,999	1579	1316
**G3116-35 k-mer**	366,215,814	79,936	13,528	1624	1372
**G3116 Merged**		**117,969**	**22,045**	**1783**	**1525**

Transcriptome assembly statistics for *T. monococcum ssp*. *monococcum* (DV92) and *T. monococcum ssp. aegilopoides* (G3116) generated by Velvet/Oases. The statistics describe the sequence input to the assembler and the number of assembled transcripts and relative transcript length in base pairs (bp). The merged assembly is a feature of Oases that merges transcript isoforms into putative gene loci.

### Comparisons with the Triticeae genomes

**Figure 1:**
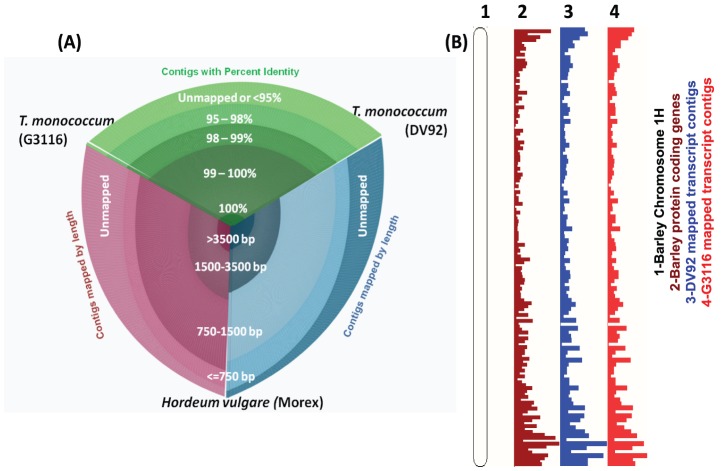
Mappings DV92 and G3116 transcripts to the sequenced *Hordeum vulgare* (barley) genome v1.0 (source: Gramene/Ensembl Plants). (A) A hive plot shows comparison between *Triticum monococcum* accessions G3116 and DV92 vs. the barley genome. (B) A density plot view of the Ensembl Plants genome browser showing barley chromosome-1H karyotype view (track-1) with annotated barley genes (track-2; maroon) and the mapped G3116 transcripts (track-3; blue) and DV92 transcripts (track-4; red).

To annotate, characterize and approximate the coverage of sequenced and assembled transcripts representing common gene loci, we compared the transcripts of DV92 and G3116 to transcripts of other plant species from Poaceae (Table 2) using BLAST [30]. *Triticum* shares a more recent common ancestor with barley than with *Brachypodium* [11], therefore, we chose the barley genome (Gramene 030312 v2.18) as the reference for further comparative analysis. Over 92% of transcripts from both DV92 and G3116 were successfully mapped to the barley genome and show broad coverage of the genome (Table 2; Figure 1). Approximately 77% of DV92 and G3116 transcripts mapped to ∼90% of the barley gene models with ≥95% percent identity (Figure 1; Tables 2 and 3). In the reciprocal BLAST analysis, we successfully mapped ∼91% of the barley gene models to the G3116 transcriptome and ∼93% of the barley transcripts to the DV92 transcriptome (Table 3).

**Table 2:** BLAST results.

Target	Query			
	DV92 (120,911)		G3116 (117,969)	
	*# hits*	*% hits*	*# hits*	*% hits*
**DV92**	-	-	116,227	98.50%
**G3116**	117,872	97.50%	-	-
***T. urartu*** (wheat A genome) ^*^	118,618	98.10%	115,498	97.90%
***T. urartu*** Transcripts^*^	102,176	84.50%	99,148	84.00%
***A. Tauschii*** (wheat D genome)^*^	120,061	99.30%	117,090	99.25%
***A. tauschii*** Transcripts^*^	104,932	86.70%	101,749	86.25%
***T. aestivum*** ** Transcripts^§^**	115,528	95.50%	113,064	95.80%
***T. aestivum*** ** Transcripts^∧^**	115,244	95.30%	112,786	95.60%
***H. vulgare*** ** genome v2.18^#^**	112,442	92.30%	109,816	93.10%
***H. vulgare*** ** Transcripts v2.16^#^**	93,369	77.20%	91,411	77.50%
***O. sativa indica*** ** ASM465 v1.16^#^**	83,775	69.30%	82,176	69.70%
***O. sativa japonica*** ** MSU6^#^**	84,836	70.20%	83,291	70.60%
***B. distachyon*** ** v1.1^#^**	88,655	73.30%	86,990	73.70%

Source: *GigaBD; #Gramene; ∧Plant GDB GenBank release 175; § DFCI release 12.0.

BLASTn (E-value 1e^−5^) nucleotide sequence comparisons of *T. monococcum ssp*. *Monococcum* (DV92) and *T. monococcum ssp. aegilopoides* (G3116) transcripts against gene models and genomes from other sequenced grass species suggesting the coverage represented in the *T. monococcum* transcriptome.

**Table 3:** The coverage and mapping of *T. urartu*, *A. tauschii* and *H. vulgare* transcripts on DV92 and G3116 transcriptomes using BLASTn (E-value 1e^−5^).

Target	Query					
	*T. urartu*		*A. tauschii*		*H. vulgare*	
	(Transcripts #34,879)^*^		(Transcripts #43,150)^*^		(Transcripts #62,240)^∧^	
	*# hits*	*% hits*	*# hits*	*% hits*	*# hits*	*% hits*
**DV92**	29,784	85.40%	35,618	82.50%	57,781	92.80%
**G3116**	29,108	83.40%	34,783	80.60%	56,609	90.90%

Source: *GigaBD; ∧Gramene.

The number of transcripts and percent of transcripts from each query that hit a transcript from DV92 and G3116 are shown.

Comparison of the DV92 and G3116 transcriptomes with the *T. urartu* (wheat A genome) and the *A. tauschii* (wheat D genome) genomes and gene models [15]–[17] suggest that ∼84% of the *T. monococcum* transcripts from both accessions mapped to the *T. urartu* gene models, while ∼86% mapped to the *A. tauschii* gene models (Table 2). 80–85% of the *A. tauschii* and *T. urartu* coding sequences matched DV92 or G3116 transcripts in a reciprocal BLASTn analysis (Table 3).

### Functional annotation

InterPro domain annotations were assigned to 54,814 DV92 transcripts and 53,627 G3116 transcripts based on analyses of putative polypeptide encoded by the longest Open Reading Frame (ORF) for a given transcript (Table S1). InterPro domain mappings provided Gene Ontology (GO) annotations for 42,931 DV92 transcripts and 41,983 G3116 transcripts. Blast2GO [31] analysis provided GO annotations for 64,950 DV92 and 61,783 G3116 transcripts (see Data Access section). Using both InterPro and Blast2GO methods, we assigned functional annotation to a total of 71,633 (59.0%) DV92 and 69,437 (58.8%) G3116 transcripts. Overall, 2,897 and 2,867 GO terms were assigned to DV92 and G3116 transcripts respectively, with 2,742 GO terms common to both.

### Differential expression of genes during photomorphogenesis

**Figure 2:**
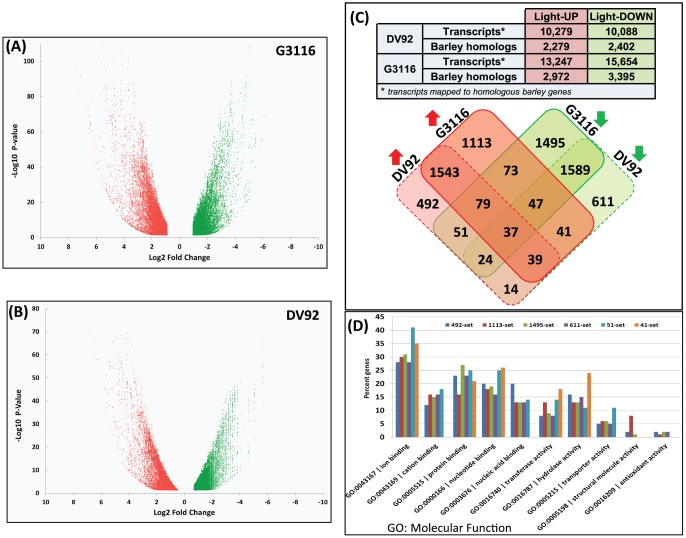
Analyses of the differentially expressed transcripts. A scatter plot of light up- regulated (red colored) and down-regulated (green colored) transcripts from G3116 (A) and DV92 (B) accessions of *T. monococcum*. Each spot represents a single transcript. (C) The table lists counts of differentially expressed transcripts from the DV92 and G3116 accessions shown in the adjacent scatter plots and their barley homologs. The four-way Venn diagram shows the distribution of barley homolog counts with reference to the mapped light up-regulated (red shaded boxes) and light down-regulated (green shaded boxes) transcripts. (D) Barley homologs from various unique sets identified in the Venn diagram (C) and their selected molecular function enrichment.

The RNA-Seq short reads from the dark-grown, etiolated (8DD) and light-exposed, green (48LL) samples were mapped against the respective transcriptomes of DV92 and G3116 to study light-regulated gene expression during photomorphogenesis. 25,742 G3116 and 23,526 DV92 transcripts show ≥2-fold change in expression (p ≤0.05) between 8DD and 48LL samples (Figure 2A and B). G3116 contains more light up-regulated and down-regulated transcripts compared to DV92 (Figure 2A and C). The differentially expressed transcripts from both accessions maps to 7,248 (30%) unique barley homologs. Henceforth, we analyzed differential expression of corresponding putative homologous *T. monococcum* genes in etiolated (8DD) and green (48LL) samples across two accessions DV92 and G3116 in a four-way comparison (Figure 2C). Compared to DV92, more than double the number of unique genes in G3116 are up- and down-regulated by light. Thirty-seven genes (Table S2) show a common profile across all four samples. This set includes homologs of light-harvesting chlorophyll B-binding protein, 3-ketoacyl-COA synthase, pyruvate kinase, tubulin beta chain, red chlorophyll catabolite reductase and cellulose synthase-like protein (Table S2). Interestingly, unique set of fifty-one genes show increased expression in DV92, but decreased expression in G3116 in response to light (Figure 2C). This set includes homologs of rubisco activase, brassinosteroid-6-oxidase, 3-ketoacyl-CoA-synthase, histone H2A, SEC-C motif-containing protein, ATP-dependent *clp* protease ATP-binding subunit, heat shock protein 90 and cpn60 chaperonin family protein (Table S2). Conversely, a set of forty-one genes shows decreased expression in DV92 but increased expression in G3116 in response to light (Figure 2C). This set includes homologs of germin-like protein 1, plastid transcriptionally active 13, Tetratricopeptide repeat (TPR)-like superfamily protein and CAX interacting protein 1 (Table S2).

For each set of differentially expressed genes (Figure 2C), enrichment of a selected GO molecular function categories is shown in Figure 2D. We found that the 41- and 51-gene sets show enrichment for proteins that are likely to have ion and cation binding, nucleotide binding and transfer activities. The 41-gene set has a greater percentage of hydrolases, whereas, the 51-gene set contains a greater percentage of transporters (Figure 2D). Among the light up-regulated genes common to both DV92 and G3116, we found enrichment of genes encoding for structural components of cell envelopes, proteins involved in anatomical structure formation and proteins associated with cellular component biogenesis, having cellular component location ‘plastid’ (GO:0009536) or ‘intracellular organelle’ (GO:0043229), and enrichment of gene products targeted to ‘thylakoid’ (GO:0009579). Other categories of genes that show increased expression after exposure to light include components of carbohydrate metabolism, namely, the ‘oligosaccharide metabolism’ (GO:0009311), cell wall remodeling (GO:0004553; glycosyl hydrolases), and ‘post-translational protein modification’ (GO:0043687). The light down-regulated genes were associated with the biological process ‘phosphate metabolic process (GO:0006796) with enrichment for ‘nucleotide diphosphatase activity’ (GO:0004551) (Table S3).

In DV92, transcripts encoding red (phytochrome) and blue (cryptochrome) light receptor proteins are down-regulated by 2-fold or more, whereas, orthologous transcripts in G3116 are up-regulated by 2-fold or more during photomorphogenesis (Table S1). A small subset of DV92 and G3116 transcripts mapped to genes with known homologs in plants exhibit differential expression during photomorphogenesis (Table S4). The light-induced genes include *lhcb* coding for chlorophyll a/b binding proteins, *Elongated hypocotyl 5* (*HY5*) coding for a positive regulator of photosynthesis associated nuclear genes, *rbcs* coding for ribulose bisphosphate carboxylase small subunit, homologs of rice *YGL138(t)* gene involved in chloroplast development [32], genes coding for mitochondrial transcription termination factor, late embryogenesis abundant protein LEA, and those coding for Rossmann-like alpha/beta/alpha sandwich fold containing protein (Table S4). Notably, homologs of gene coding for ABA 8′-hydroxylase activity associated with germination are significantly light up-regulated in G3116 but not in DV92. The light down-regulated genes include homologs of wheat *Rht-B1* DELLA protein, a nuclear repressor of gibberellin response, and *TaIAA1*, a primary auxin-response gene [33].

### Developing genetic marker resources from the sequenced transcriptome

Molecular genetic markers are very useful for the analysis of genetic variation and heritable traits. Well established genotyping methods, such as high-throughput genotyping-by-sequencing (GBS) and chip-based methods using genomic DNA facilitate the interrogation of SNP and SSR markers. Similarly, large RNA-Seq data sets can be mined for molecular marker sites [27], which may then be used for genetic trait mapping, diversity analysis and marker-assisted selection in plant breeding experiments. This method permits future systems-level studies to explore the integrated analysis of gene function, expression, and the consequence of sequence variation on gene structure and function.

### Identification of SSR marker loci

**Figure 3:**
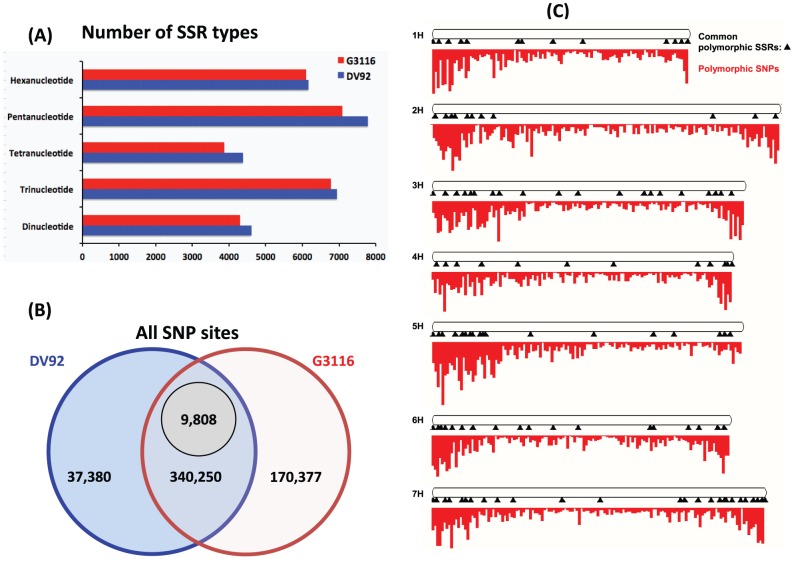
Genetic marker discovery. Polymorphic sites identified in the transcriptome of DV92 (blue) and G3116 (red). (A) Number of SSR identified in the transcriptomes. (B) Number of SNPs identified in the two genotypes by aligning against the sequenced barley reference genome. 9,808 out of 340,250 common SNP sites have polymorphism between DV92 and G3116. (C) Mapping of common, variable 9,808 SNP and 148 SSR sites identified in the DV92 and G3116 transcriptomes on the karyotype view of the reference barley genome hosted by the Ensembl Plants. The SNP sites are shown as red colored density plot and SSR sites are depicted as black triangles along the length of the respective barley chromosomes.

We mined the DV92 and G3116 transcriptome assemblies for di-, tri-, tetra-, penta-, and hexa-nucleotide SSRs with a minimum of 8, 6, 4, and 3 repeat units, respectively. We identified 29,887 SSR sites in 22,019 unique DV92 transcripts and 28,122 SSR sites in 20,727 unique G3116 transcripts (Figure 3A; Table S5). 3,413 transcripts orthologous between DV92 and G3116 contain identical SSRs, whereas 703 DV92 and G3116 orthologous transcripts contain variable-length SSRs. Some of these 703 sites may represent duplicate SSRs found in transcripts that map to the same or overlapping locus; therefore we aligned our assembled transcripts to the barley genome and identified 148 unique barley gene loci that harbor the variable SSR-containing sequence (Figure 3C). We experimentally verified a small number of SSRs for genotyping the DV92 and G3116 accessions (data not shown), though a majority of the markers will require experimental validation before they can be used.

### Identification of SNP marker loci

To identify single nucleotide polymorphism (SNP) sites across the DV92 and G3116 transcriptomes, we used SOAPsnp [34] to align and identify the raw *T. monococcum* sequence reads against the barley genome. We identified 510,627 SNPs with an average of one SNP per 3600 bp of the assembled barley genome. Of these, 170,377 SNP sites were unique to G3116, and 37,380 SNP sites were unique to DV92 (Figure 3B). More than 50% of the SNP sites (330,444) are present in both the DV92 and G3116 accessions. Of these common sites, 9,808 SNP sites were identified with different alleles for DV92 and G3116. These 9,808 SNP sites show a uniform distribution along the barley genome (Figure 3C), thus holding potential utility as genetic markers in wheat breeding programs. These 9,808 SNP sites are present in 5,989 unique protein coding genes, which include a subset of 4,935 GO-annotated genes (Table S6) and 2,543 differentially expressed genes. A greater number of nucleotide transitions were also discovered in DV92 when compared to G3116, which had more transversions (Table S7). In order to address the biological relevance of these SNPs, we predicted the potential effects of the variants and identified a diverse set of consequences on the transcript's structure, splicing and protein coding features with reference to the barley genome and annotated gene models (Table 4). Notably, we identified over 300,000 downstream variants, ∼200,000 missense variants, 10,000-18,000 transcript splice site mutations, and more than 400 sites with a gain in stop codons (Table 4). Unique DV92 and G3116 SNPs are distributed across variance consequence categories in similar proportions to combined SNPs (Table 4).

**Table 4:** Prediction of SNP variant consequence with reference to the annotated barley genome.

Predicted variant effect	Number of SNP sites with consequences		Unique	
	DV92	G3116	DV92	G3116
3 prime UTR variant	131,758	165,696	6,918	9,022
5 prime UTR variant	86,389	127,854	4,450	9,371
coding sequence variant	21,545	30,920	2,422	3,704
downstream gene variant	328,112	440,765	19,120	26,060
initiator codon variant	364	507	22	49
initiator codon variant, splice region variant	6	8	none	None
intergenic variant	35,753	54,722	2,682	24,136
intron variant	46,901	111,413	4,717	14,217
missense variant	198,794	258,081	9,929	17,763
missense variant, splice region variant	1,145	1,866	89	188
non coding exon variant, nc transcript variant	7	11	1	1
splice acceptor variant	10,094	18,609	572	2,103
splice donor variant	18,433	34,503	1,145	3,985
splice region variant, 3 prime UTR variant	681	962	25	51
splice region variant, 5 prime UTR variant	685	1,137	50	80
splice region variant, coding sequence variant	136	272	15	39
splice region variant, downstream gene variant	2	3	none	None
splice region variant, intron variant	31,692	63,891	2,270	8,074
splice region variant, synonymous variant	3,500	5,083	176	448
stop gained	462	732	40	62
stop gained, splice region variant	4	8	none	2
synonymous variant	451,169	538,115	18,687	29,046

SNP Variant Consequence Prediction based on the *T. monococcum* SNPs identified by aligning the sequenced reads from DV92 and G3116 to the reference barley genome and the barley gene models (v1.0) available from Ensembl Plants database. Listed variant effect types are based on the categories adopted by the Ensembl Plants database.

## Discussion

This study provides the *de novo* assembled transcriptomes of two *T. monococcum* sub-species, representing the domesticated accession DV92 and the wild accession G3116. High-throughput RNA-Seq technology, bioinformatics tools and publicly available databases enabled higher quality transcriptome assemblies of these diploid wheat varieties, both of which are closely related to the wheat A-genome progenitor *T. urartu*. However, approximately 15% of the DV92 and G3116 transcriptomes do not map to the *T. urartu* and *A. tauschii* (progenitor of the wheat D genome) gene models (Table 3). We compared these unmapped *T. monococcum* transcripts against the barley genome and found 4,954 DV92 and 5,362 G3116 transcripts bear homology to 2,607 barley genes, suggesting that these genes have not been annotated in the published wheat A and D genomes [15]–[17]. Furthermore, comparison of the *T. monococcum*, *T. urartu* and barley gene models also revealed other disparities. For example, gene models for the *T. urartu* gene TUIUR3_02586-T1 lack exon-4, 3′ and 5′ UTRs and potentially unspliced introns when compared to the barley homolog MLOC_59496. In our analysis, multiple *T. monococcum* transcript isoforms aligned with the barley homolog MLOC_59496 support the barley gene model (Figure S2) and thus provide empirical evidence for the missing features in *T. urartu* gene TUIUR3_02586-T1 (Figure S3). Our findings demonstrate the utility of the *T. monococcum* transcriptome data in enriching and improving Triticeae genome annotation, including the recently published A and D genomes.

To our knowledge, this study is the first to provide the relative expression of transcript isoforms (Figure 2, Table S1) in both etiolated seedlings and light-exposed green seedlings of cultivated spring accession DV92 and wild winter accession G3116 of *T. monococcum* (Figure S4). In order to preserve the granularity of the transcript isoform-based expression profile, we avoided projecting a weighted expression profile of the genes. This allowed us to identify a greater number of differentially expressed transcripts in G3116 (Figure 2A). However, for simplicity, the four-way Venn diagram (Figure 2C) was constructed to show comparison between the light up- and down-regulated genes from the two accessions.

In general, the transcriptomes of both DV92 and G3116 suggest up-regulation of the genes involved in chloroplast biogenesis, photosynthesis and carbohydrate metabolism, such as the homologs of *Elongated hypocotyl 5* (*HY5), YGL138(t)* [32], [35] and photosystem II chlorophyll a/b-binding protein *lhcb* (Table S4). In addition, differentially expressed transcripts encoding for mitochondrial transcription termination factor-like protein (mTERF), late embryogenesis abundant protein (LEA) and Rossmann-like alpha/beta/alpha sandwich fold containing protein family members were found to be light up-regulated (Table S4). In humans, the mitochondrial transcription termination factor attenuates transcription from the mitochondrial genome, up-regulates the expression of 16S ribosomal RNA, and has high affinity for the tRNA^Leu(UUR)^ gene [36]–[38]. The Arabidopsis mTERF gene family members are known to play roles in organelles; for example, *SUPPRESSOR OF HOT1-4 1 (SHOT1)*, a mitochondrial protein, is involved in heat tolerance and regulation of oxidative stress [39], *SINGLET OXYGEN-LINKED DEATH ACTIVATOR10 (OLDAT10)*, a plastid protein, activates retrograde signaling and oxidative stress, and *BELAYA SMERT* (*BSM*) regulates plastid gene expression [40]. The mTERF domain containing proteins from both the DV92 and G3116 accessions showing light up-regulation are predicted to be chloroplast proteins (TargetP value ∼0.9) (Figure S5). To our knowledge, this is the first report of light up-regulation of wheat gene family members encoding mTERF, LEA and Rossmann-like alpha/beta/alpha sandwich fold containing proteins.

Other proteins that show light-induced differential regulation are involved in phytohormone metabolism and signaling. Transcripts homologous to *T. aestivum Rht-B1* that code for a DELLA protein were down-regulated by light [41]. DELLA proteins are repressors of gibberellin (GA) signaling and act immediately downstream of GA receptor. When GA synthesis is induced by light, the binding of GA to its receptor causes degradation of DELLAs via the ubiquitin-proteasome pathway [42]. GA is a hormone that is well known to promote seed germination in addition to participating in other parts of the plant life cycle. DELLAs have also been suggested to mediate interaction between GA and abscisic acid (ABA) pathways, as one of its targets, *XERICO*, is known to regulate ABA metabolism [42]. The levels of transcripts homologous to ABA 8′-hydroxylase were significantly higher in G3116 relative to DV92. ABA 8′-hydroxylase degrades ABA, a hormone involved in dormancy [43]. Degradation of ABA results in a decreased ABA-to-GA ratio resulting in the breaking of dormancy [44]. ABA 8′-hydroxylase activity may be one of the difference between winter and spring varieties. Conversely, increased levels of transcripts homologous to gene encoding for brassinosteroid-6-oxidase were found in DV92 in response to light, but not in G3116. Transcripts homologous to *TaIAA1*, an early auxin-response gene from wheat [33], were down-regulated by light in both DV92 and G3116, which is consistent with the previous report [33]. In addition to auxin, the *TaIAA1* gene is also induced by brassinosteroids [33]. Several genes showed accession-specific expression profile, such as the 51 and 41 gene sets (Figure 2C, Table S2), which may reflect differences in anatomical features and the plant's response to its immediate environment. For instance, the levels of transcripts homologous to rice *germin-like protein 1* show decrease in DV92 but increase in G3116 in light-exposed seedlings. The *germin-like protein-1* in rice has been shown to play a role in the regulation of plant height and disease resistance [45]. Transcripts homologous to genes coding for heat shock protein 90 and cpn60 chaperonin family protein increase in DV92, but decrease in G3116 in response to light (Table S2). Changes in the expression levels of transcripts encoding components of hormone biosynthesis, signaling and protein targets suggest that photomorphogenesis is a carefully orchestrated interplay of both developmental signals (often genotype-specific) and light response.

We identified over 500,000 SNP sites and approximately 22,000 SSR/microsatellite sites in the transcriptome assemblies of *T. monococcum*. Of these, 9,808 SNP and 148 SSR sites are common polymorphic sites in both accessions. The 9,808 SNPs overlap 2,543 barley genes that show light mediated up- and down-regulation of homologous transcripts in *T. monococcum*. A few notable genes in this differentially expressed set include (Figure S6 and Table S8) the light down-regulated protein coding genes for CASP-like membrane protein, Xyloglucan endo-transglycosylase activity, Auxin-responsive family protein and a novel protein carrying the DUF1644 domain. Whereas, the light up-regulated protein coding genes includes, photosystem-I subunit PSAK, PSAH, Ribulose-1,5-bisphosphate carboxylase (RUBISCO) small subunit RBCS, Chlorophyll a/b binding protein LHCB, Mitochondrial transcription termination family member and novel uncharacterized proteins (Figure S6 and Table S8). Our data suggest that 170,377 SNPs is unique to G3116 and 37,380 SNPs is unique to DV92 (Figure 3B); this provides an opportunity to study the wild winter and cultivated spring habits of the two accessions in greater detail. The SNP and SSR genetic sites identified in our dataset, along with those identified in other genetic populations [46] and wheat projects [47], will provide useful marker resources for fine mapping experiments and marker-assisted wheat breeding programs.

Along with the *T. monococcum* transcriptomes from two accessions, we have provided additional genomic and genetic resources including their functional annotations, differential gene expression analyses and potential SNPs and SSRs, which can be used to explore Triticeae genome diversity, co-expression networks involved in photomorphogenesis and to develop stochastic and metabolic networks [22], [48], [49]. In addition, these resources can be used to identify novel genes, transcript models and eQTLs, and to study plant's adaptation to diverse climatic conditions, impacts of domestication on crop plants and evolution of novel genes.

## Methods

### Plant material and growth conditions

Seeds of the *Triticum monococcum* ssp. monococcum accession DV92, a cultivated spring wheat, and *Triticum monococcum* ssp. aegilopoides accession G3116, a wild winter wheat, were sown into sunshine mix (Sun Gro Horticulture, Agawam, MA, USA). The trays were watered thoroughly and were shifted (in the evening hours) to a dark growth chamber set to cycle temperature between 20°C for 12 hours (8am–8pm) and 18°C for the next 12 hours (8pm–8am). The seedlings were grown in the dark for next 8 days and the soil was kept moist by gently spraying with water every 72 hours. Seeds were not vernalized prior to sowing. Germination was observed within two days for both accessions. The first set of dark-grown seedlings shoot samples (8DD), consisting of three replicate from each accession, were collected at the end of day-8 under green light. (8DD). On day-9 at 10 am, continuous light (120 µmol/m^2^/sec at soil surface) was started for 48 hours (48LL) and a second set of seedling shoot samples (48LL), consisting of three replicates from each accession, were collected at the end of 48 hours of treatment on day 11. Each replicate contained shoots of three seedlings of similar height (Figure S4). Harvested samples were immediately frozen in liquid nitrogen and stored at −80°C.

### Sample preparation for Illumina sequencing

Total RNA from frozen seedling shoot sample was extracted using RNA Plant reagent (Invitrogen Inc., USA), RNeasy kits (Qiagen Inc., USA), and treated with RNase-free DNase (Life Technologies Inc., USA) as previously described [27], [50]. The mRNA concentration, quality were determined using ND-1000 spectrophotometer (Thermo Fisher Scientific Inc., USA) and Bioanalyzer 2100 (Agilent Technologies Inc., USA). Samples were prepared using the TruSeq RNA Sample Preparation Kits (v2) and sequenced on the Illumina HiSeq 2000 instrument (Illumina Inc., USA) at the Center for Genomic Research and Biocomputing, Oregon State University.

### *De novo* transcriptome assembly and annotation

Illumina sequences were processed for low quality at an error rate of 0.00001, parsed for index sequences and pairs, and filtered and trimmed using customized Perl scripts. FASTQ file generation and removal of low quality reads were performed by CASAVA software v1.8.2 (Illumina Inc.). The high-quality sequences used in the assembly process included 435,806,374 and 366,215,814 paired-end 101 bp reads for DV92 and G3116 respectively (Table 1). The samples were assembled with Velvet (Velvet v1.2.08), which uses De Bruijn graphs to assemble short reads [51]. An assembly of 31 and 35 k-mer length was performed separately for both the DV92 and G3116 reads. The assemblies generated by Velvet were analyzed using Oases (Oases v0.2.08), which was developed for the *de novo* assembly of transcriptomes [26], and uses the read sequence and pairing information to produce transcript isoforms.

Similarity searches were conducted with BLASTn [30] (E-value < = 1e^−5^) using assembled transcripts as a query against gene model sequence databases of other species of grasses with sequenced genomes, namely, hexaploid wheat (*T. aestivum*) transcripts (DFCI release 12.0), *T. aestivum* (Plant GDB GenBank release 175), barley (*Hordeum vulgare*) transcripts (Gramene v.2.16), barley genome (Gramene v.2.16), *Oryza sativa* spp. indica (Gramene ASM465v1.16), *Oryza sativa* spp. japonica (Gramene MSU6.16), *Brachypodium distachyon* transcripts (Gramene v.0.16), and the *Brachypodium distachyon* genome (NCBI). *T. monococcum* transcripts were functionally annotated using a combined approach based upon functional motif analysis and sequence homology. Transcripts were translated into the longest predicted open reading frame (ORF) peptide sequences using the ORFPredictor web application [52] and resulting proteins assigned InterPro identifiers using InterProScan v4.8 [53], [54]. These InterPro assignments were also mapped to Gene Ontology (GO) terms. Additionally, we did Blast2GO analysis [31] of *T. monococcum* transcripts to transfer GO annotations from functionally annotated genes in non-wheat genomes. A BLASTx search (E-value ≤1e^−2^ and percent identity ≥90%) was performed to identify highly homologous sequences against the NCBI GenBank non-redundant protein database. The resulting best hits with GO annotations were used to project similar GO assignments [55], [56] to *T. monococcum* transcripts. GO annotations from both methods were combined and duplicated annotations were removed to produce non-redundant gene ontology annotation files for *T. monococcum* DV92 and G3116. The AgriGO Analysis Toolkit [57] was used to identify statistically-enriched functional groups. This method includes a Fisher's exact test with a Yekutieli correction for false discovery rate calculation. Significance cutoffs included a P-value of 0.05 and a minimum of 5 mapping entries per GO term.

### Genetic marker development

The assemblies of DV92 and G3116 were mined for SSRs using Perl code from the Simple Sequence Repeat Identification Tool (SSRIT; [58]; http://archive.gramene.org/db/markers/ssrtool). We identified di-, tri-, tetra-, penta-, and hexa-nucleotide SSRs with a minimum of 8, 6, 4, 3, and 3 repeat units, respectively. We then used custom Perl scripts to identify orthologous DV92 and G3116 transcripts containing common SSRs.

An alignment database was generated using SOAP's 2bwt-builder with the barley genome (version 030312v2). Illumina sequences (FASTQ formatted) of length 51 bp were processed and aligned through SOAP (Version: 2.20) [59] with default options. Alignment data was then separated into different text files based on the chromosome of the hit sequence and each chromosome alignment file was sorted based on hit start position. After separation and sorting, data was processed through SOAPsnp (version 1.02) [34] to identify single nucleotide polymorphisms (SNPs). SOAPsnp was run using standard options for a diploid genome as stated in the documentation. SOAPsnp output files were then reformatted to VCF output, a community standard format developed by the 1000 Genomes project (http://www.1000genomes.org/wiki/Analysis/Variant%20Call%20Format/vcf-variant-call-format-version-41) to make them more accessible for analysis by other downstream programs. To call a SNP, values for novel homozygous prior probability and novel heterozygous prior probability were set at 0.0005 and 0.0001, respectively. The transition/transversion ratio was set to 2∶1 in prior probability. The rank sum test was enabled to give heterozygous prior probability further penalty if reads did not have the same sequencing quality for better SNP calling. A maximum read length of 51 bp was used. We used the Ensembl Plants API Effect Predictor tool [60] to infer potential consequences of the SNP variants.

### Gene expression analysis

We used CASHX v2.3 to align the DV92 and G3116 reads to their respective transcriptome assembly [61]. Indexed reads were used for each replicate for both dark and light comparisons of DV92 and G3116. We then used Edge R-package (v. 2.0.3) [62] to conduct differential gene expression analysis. We identified differentially expressed transcripts with a significance of P-value cutoff/FDR corrected P-value of 0.05. We also further filtered the differentially expressed genes by 2-fold cutoffs and those identified to be differentially expressed by the EdgeR. Principal components analysis (PCA) multidimensional scaling (MDS), and correlation matrix algorithms were used to assess and visualize a cross-sample comparisons. Both analyses show clustering based upon RPKM values for all genes among all replicates. The results, as expected, show four separate visualized clusters (DV92 light and dark replicates and G3116 light and dark replicates; Figure S7–9).

### Data Access

Sequence files, assemblies, annotation files, SNP, SSR, transcript alignments, gene expression, network data files and results are available from the project's data site [63] ScholarsArchive at Oregon State University (http://hdl.handle.net/1957/47475). The transcriptome data are being integrated in the Barley Genome Browser available from the Ensembl Plants database (http://plants.ensembl.org). The data are also being provided to the small grains database GrainGenes (http://www.graingenes.org). The raw sequence files were submitted to the National Center for Biotechnology Information (NCBI) Sequence Read Archive under the accessions SRX283514/SRR924098 (DV92) and SRX257915/SRR922411 (G3116).

## Supporting Information

Figure S1 **The frequency distribution of transcripts of varying size (bp: base pair) in the *de novo* transcriptome assemblies of DV92, G3116 and the annotated transcriptomes of barley and wheat *T. urartu*.**
(TIFF)

Figure S2 **A view of the Ensembl Plants barley genome browser showing the comparison between the models of barley gene MLOC_59496 and the homologous *T. monococcum* gene models derived from DV92 and G3116 transcriptomes.** This alignment was generated using the Exonerate software package by allowing for gapped alignments (introns). The red arrows depict intron retention events and the blue arrow depicts intron-3 in the annotated barley gene model. Our data support barley MLOC_59496 gene model, including its 3′ and 5′ untranslated regions shown by open blocks.
(TIFF)

Figure S3 **A view of the Ensembl Plants *T. urartu* genome browser showing the comparison between the *T. urartu* gene TUIUR3_02586-T1 model and the homologous *T. monococcum* gene models derived from DV92 and G3116 transcriptomes.** This alignment was generated using the Exonerate software package by allowing for gapped alignments (introns). Our models show retention of introns (red arrows) in a couple of *T. monococcum* gene models, and the presence of an exon -4 (same as exon-3 in the barley model shown in figure-S2) missed in the *T. urartu* genome annotation (the dotted-line box). Our data do not support the presence of exon-3 in (blue arrow) in the annotated *T. urartu* gene TUIUR3_02586-T1.
(TIFF)

Figure S4 **Seedling samples used for generating the transcriptomes of wheat accessions DV92 (left panel) and G3116 (right panel).**
(TIFF)

Figure S5 **TargetP analysis of the DV92 and G3116 peptides bearing the Mitochondrial transcription termination factor-related domain.** The proteins were predicted to be targeted to chloroplast (cTP) with a high confidence score of ∼0.9. Both peptides were predicted to have a transit peptide length (Tplen) of 78aa.
(TIFF)

Figure S6 **The line plot display of expression level in RPKM log2 values of transcripts that were grouped into light down regulated and light up-regulated co-expressed clusters (Figure 3 and 4) and have overlapping SNPs from the 9,808 SNP set.** The table on the right shows homologous barley gene, functional annotation and the SNP variant effect on the transcript structure and/or function.
(TIFF)

Figure S7 **Principal component analysis (PCA) analysis of RNA-Seq reads.**
(TIFF)

Figure S8 **Multidimensional scaling (MDS) analysis of RNA-Seq reads.**
(TIFF)

Figure S9 **Correlation matrix analysis of RNA-Seq reads.**
(TIFF)

Table S1 **Expression profiles of assembled transcripts from DV92 and G3116.** It is a zip file with two tab-delimited files called Table S1a.txt with DV92 gene expression data and Table S1b.txt with G3116 gene expression data.
(ZIP)

Table S2 **List of barley homologs clustered in a four-way Venn diagram (Figure 2C)**
(XLSX)

Table S3 **Enrichment of the Gene Ontology-based functional annotation of the barley homologs clustered in a four-way Venn diagram (Figure 2C).**
(XLSX)

Table S4 **A short list of transcripts mapped to known and novel genes along with their expression datasets from the DV92 and G3116 accession.**
(XLSX)

Table S5 **Counts of Simple Sequence Repeats identified in the Triticum monococcum transcriptome data.**
(XLSX)

Table S6 **Enrichment of the Gene Ontology based functional annotation of the barley homologs overlapping the 9,808 SNP sites that had a different allele for DV92 and G3116 with reference to barley allele.**
(XLSX)

Table S7 **Number of transitions and transversions resulting from SNP analysis with reference to the allele from the barley genome.**
(XLSX)

Table S8 **A list of DV92 and G3116 transcripts homologous to the barley genes overlapping the 9,800 SNP set.** Table includes DV92 and G3116 transcript IDs, homologous barley gene ID, RPKM values, respective p-value scores, putative gene function annotation and the resultant SNP variant effect with reference to the barley gene models.
(XLSX)
