# Correction: *De Novo* Transcriptome Assembly and Analyses of Gene Expression during Photomorphogenesis in Diploid Wheat *Triticum monococcum*

**DOI:** 10.1371/journal.pone.0105275

**Published:** 2014-08-01

**Authors:** The PLOS ONE Staff

The affiliation for the last author is incorrect. Pankaj Jaiswal is not affiliated with #4 but with #3. The correct affiliations for this author are:

1 Department of Botany and Plant Pathology, Oregon State University, Corvallis, Oregon, United States of America.

3 Center for Genome Research and Biocomputing, Oregon State University, Corvallis, Oregon, United States of America.
