# Supplementary figures and images for: De Novo Transcriptome Assembly and Analyses of Gene Expression during Photomorphogenesis in Diploid Wheat Triticum monococcum
Source: PLoS One. 2014 May 12;9(5):e96855. doi: 10.1371/journal.pone.0096855 (PMC4018402; doi:10.1371/journal.pone.0096855)

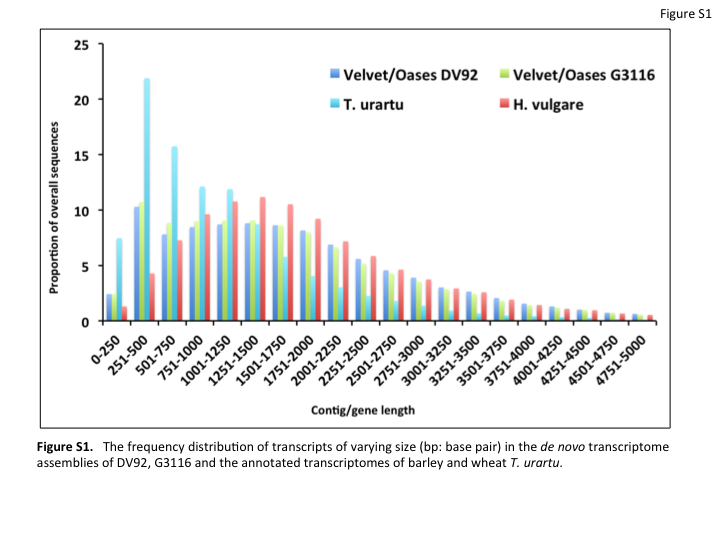

Supplement: Figure S1 — The frequency distribution of transcripts of varying size (bp: base pair) in the de novo transcriptome assemblies of DV92, G3116 and the annotated transcriptomes of barley and wheat T. urartu. (TIFF) [file pone.0096855.s001.tiff]

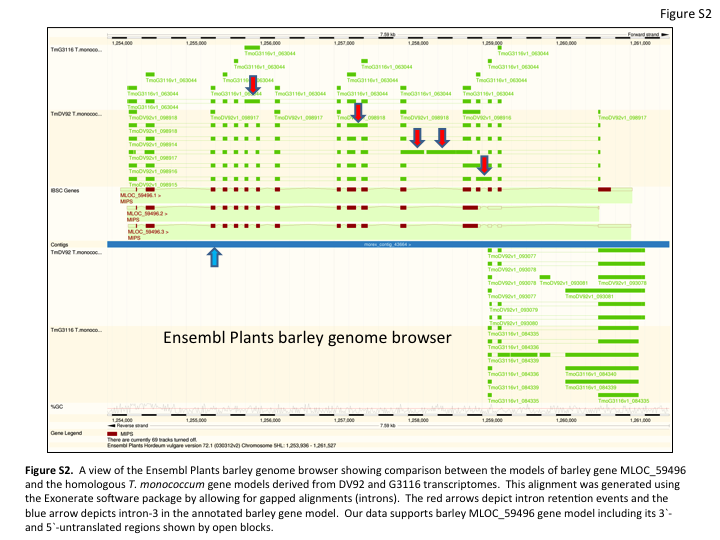

Supplement: Figure S2 — A view of the Ensembl Plants barley genome browser showing the comparison between the models of barley gene MLOC_59496 and the homologous T. monococcum gene models derived from DV92 and G3116 transcriptomes. This alignment was generated using the Exonerate software package by allowing for gapped alignments (introns). The red arrows depict intron retention events and the blue arrow depicts intron-3 in the annotated barley gene model. Our data support barley MLOC_59496 gene model, including its 3′ and 5′ untranslated regions shown by open blocks. (TIFF) [file pone.0096855.s002.tiff]

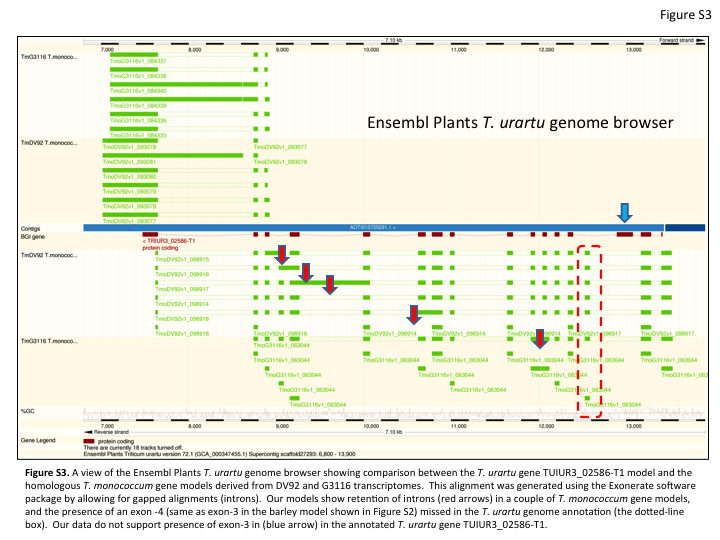

Supplement: Figure S3 — A view of the Ensembl Plants T. urartu genome browser showing the comparison between the T. urartu gene TUIUR3_02586-T1 model and the homologous T. monococcum gene models derived from DV92 and G3116 transcriptomes. This alignment was generated using the Exonerate software package by allowing for gapped alignments (introns). Our models show retention of introns (red arrows) in a couple of T. monococcum gene models, and the presence of an exon -4 (same as exon-3 in the barley model shown in figure-S2) missed in the T. urartu genome annotation (the dotted-line box). Our data do not support the presence of exon-3 in (blue arrow) in the annotated T. urartu gene TUIUR3_02586-T1. (TIFF) [file pone.0096855.s003.tiff]

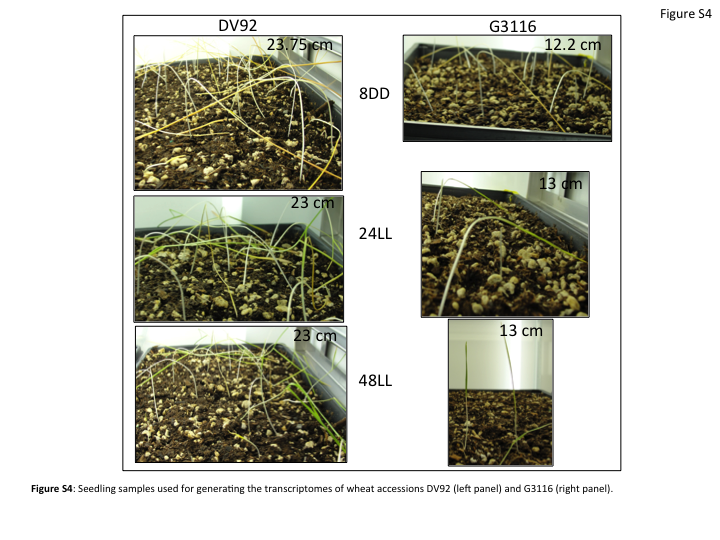

Supplement: Figure S4 — Seedling samples used for generating the transcriptomes of wheat accessions DV92 (left panel) and G3116 (right panel). (TIFF) [file pone.0096855.s004.tiff]

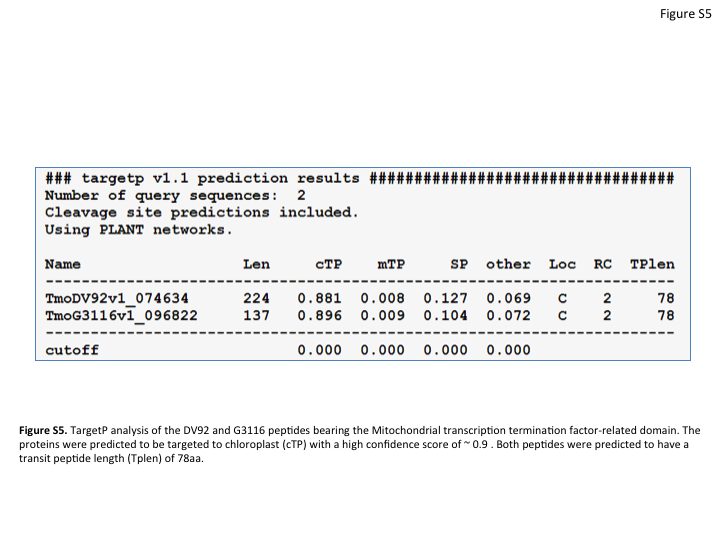

Supplement: Figure S5 — TargetP analysis of the DV92 and G3116 peptides bearing the Mitochondrial transcription termination factor-related domain. The proteins were predicted to be targeted to chloroplast (cTP) with a high confidence score of ∼0.9. Both peptides were predicted to have a transit peptide length (Tplen) of 78aa. (TIFF) [file pone.0096855.s005.tiff]

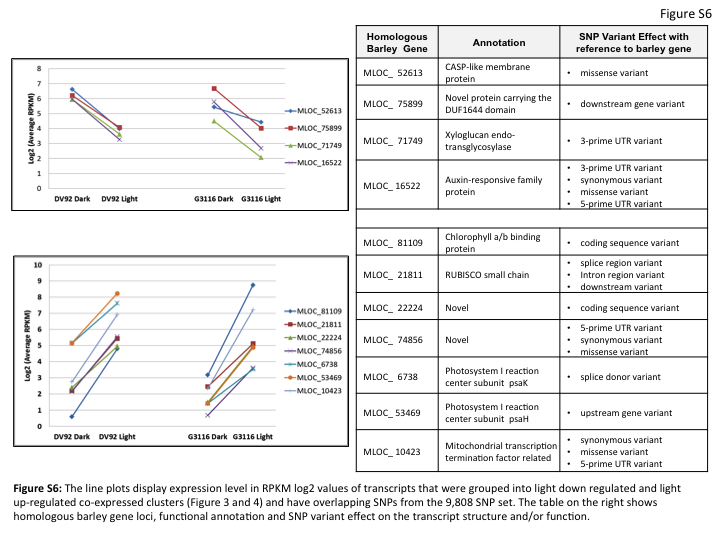

Supplement: Figure S6 — The line plot display of expression level in RPKM log2 values of transcripts that were grouped into light down regulated and light up-regulated co-expressed clusters (Figure 3 and 4) and have overlapping SNPs from the 9,808 SNP set. The table on the right shows homologous barley gene, functional annotation and the SNP variant effect on the transcript structure and/or function. (TIFF) [file pone.0096855.s006.tiff]

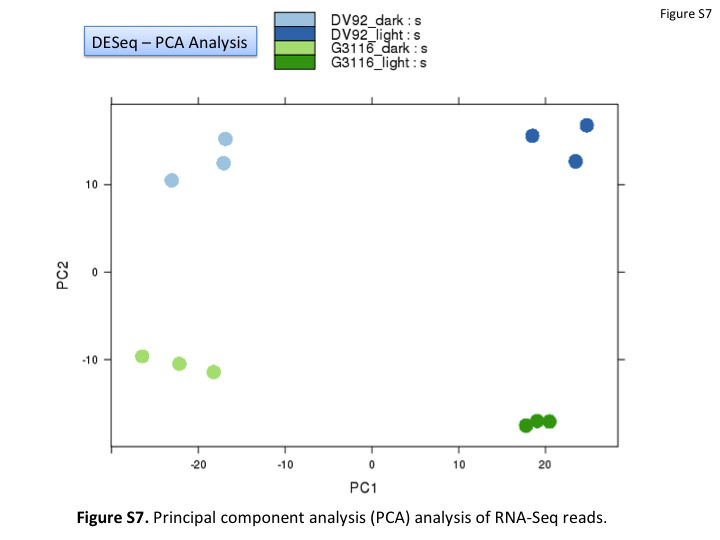

Supplement: Figure S7 — Principal component analysis (PCA) analysis of RNA-Seq reads. (TIFF) [file pone.0096855.s007.tiff]

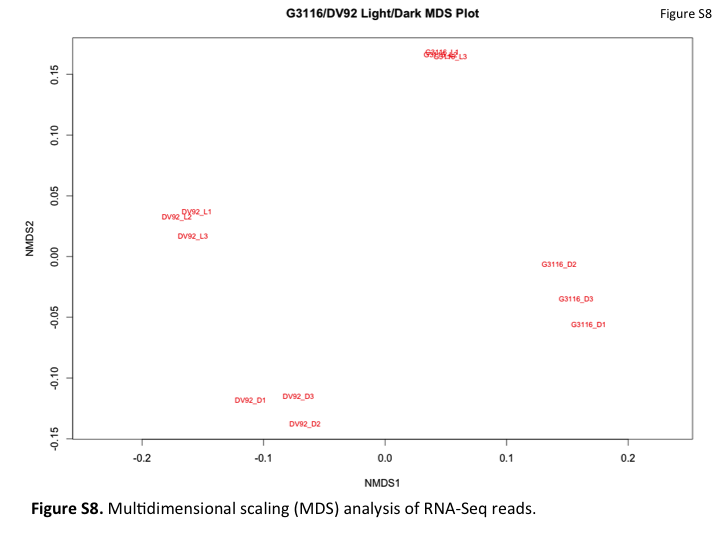

Supplement: Figure S8 — Multidimensional scaling (MDS) analysis of RNA-Seq reads. (TIFF) [file pone.0096855.s008.tiff]

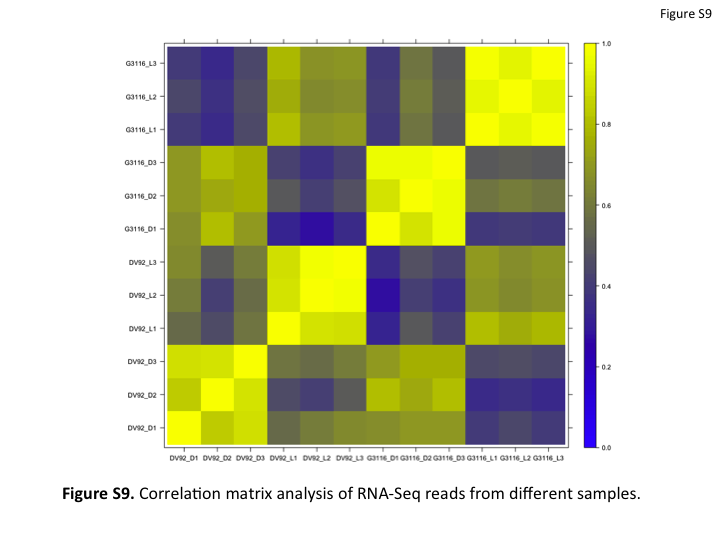

Supplement: Figure S9 — Correlation matrix analysis of RNA-Seq reads. (TIFF) [file pone.0096855.s009.tiff]
